# In Situ Microgravimetric Study of Ion Exchanges in the Ternary Cu-In-S System Prepared by Atomic Layer Deposition

**DOI:** 10.3390/ma13030645

**Published:** 2020-02-01

**Authors:** Harold Le Tulzo, Nathanaelle Schneider, Frédérique Donsanti

**Affiliations:** 1 Institut Photovoltaïque d’Ile de France (IPVF), 18 boulevard Thomas Gobert, 91120 Palaiseau, France; harold.letulzo@gmail.com; 2 French Environment and Energy Management Agency (ADEME), 20 avenue du Grésillé, 49004 Angers, France; 3 CNRS, Institut Photovoltaïque d’Ile de France (IPVF), UMR 9006, 18 Boulevard Thomas Gobert, 91120 Palaiseau, France; 4 EDF Research & Development, 18 Boulevard Thomas Gobert, 91120 Palaiseau, France

**Keywords:** ALD, sulfide materials, thin films, QCM, CIS, exchange mechanisms

## Abstract

Reaction mechanisms during the growth of multinary compounds by atomic layer deposition can be complex, especially for sulfide materials. For instance, the deposition of copper indium disulfide (CuInS_2_) shows a non-direct correlation between the cycle ratio, the growth per cycle of each binary growth cycles, i.e., Cu_x_S and In_2_S_3_, and the film composition. This evidences side reactions that compete with the direct Atomic Layer Deposition (ALD) growth reactions and makes the deposition of large films very challenging. To develop a robust upscalable recipe, it is essential to understand the chemical surface reactions. In this study, reaction mechanisms in the Cu-In-S ternary system were investigated in-situ by using a quartz crystal microbalance system to monitor mass variations. Pure binary indium sulfide (In_2_S_3_) and copper sulfide (Cu_x_S) thin film depositions on Al_2_O_3_ substrate were first studied. Then, precursors were transported to react on Cu_x_S and In_2_S_3_ substrates. In this paper, gas-phase ion exchanges are discussed based on the recorded mass variations. A cation exchange between the copper precursor and the In_2_S_3_ is highlighted, and a solution to reduce it by controlling the thickness deposited for each stack of binary materials during the CuInS_2_ deposition is finally proposed.

## 1. Introduction

Atomic Layer Deposition (ALD) is a technique based on self-limited surface chemical reactions, where the precursors are injected successively in a reactor under vacuum and heated at a relatively low temperature [1,2,3,4]. By exploiting its unique properties, conformal, pinhole-free, and precisely controlled (thickness, composition and interfaces) ultrathin binary, but also multinary films, can be synthesized. In the case of multinary compounds (ternary, quaternary materials, etc.), the most common ALD approach is the so-called “supercycle synthesis strategy” that consists of alternating binary ALD cycles [5,6,7]. Among its parameters, the cycle sequence (i.e., the order of the precursor doses) and the cycle ratio (i.e., the number of cycles of one binary process vs. the total number of cycles) are essential to obtain a homogeneous film. Added to these, nucleation delays, exchange mechanisms, growth rate, and temperature window differences complicate the deposition of such multinary compounds and make the control of the process parameters crucial [5,6,7].

The sulfide class contains a wide range of materials in which syntheses by ALD have been relatively less explored compared to others, such as oxide and elementary metals. Their interests are particularly driven by the energy-related fields [8]. In particular, Copper Indium Disulfide (CuInS_2_, CIS) is a suitable material to be used as a thin absorber in photovoltaic devices (direct bandgap ≈ 1.5 eV, absorption coefficient > 10^4^ cm^−1^ for λ = 500 nm) [9,10], and CIS-type solar cells with homo- [11] or hetero- [12,13] junction architecture have been reported. CIS synthesis has been reported by various routes, such as thermal evaporation [11], co-evaporation [14], sputtering [15], electrodeposition [16], spray pyrolysis [17], spray ion layer gas reaction (spray-ILGAR) [18], atmospheric pressure spray chemical vapor deposition (APCVD) [19], and ALD [20,21]. The complexity of the ALD deposition process of CIS, as for sulfide multinary compounds in general, lies in the high propensity of the cation exchange mechanisms, the greater diffusion rates and the unintentional annealing of the labile sulfur [8,21,22,23]. Besides a deep understanding and control of the chemical process, a rigorous cleaning of the various parts of the reactor must often also be carried out to obtain a homogeneous film [21,24]. To date, two teams have mastered these conditions and reported CIS homogenous deposition by employing quartz and small scale (5 × 5 cm^2^) reactors [20,21,25].

In the present work, an upscale was attempted to deposit CIS in a larger reaction chamber (15 × 15 cm^2^) using the supercycle strategy of Schneider, et al. [17]. A non-homogeneous film composed of copper sulfide (Cu_x_S) upstream, indium sulfide (In_2_S_3_) downstream, and a Cu_x_In_y_S_2_ film in the middle of the reaction chamber was obtained. The Cu_x_In_y_S_2_ film area was inversely proportional to Cu and In concentrations in the flux direction from the inlet to the outlet. Variation of process parameters, such as the cycle ratio, the reactor temperature, or the Cu precursor pulse time, showed a shift in the position of the Cu_x_In_y_S_2_ film area but failed to widen it. Thus, to tailor a working process on a large scale, understanding of the surface chemical reactions was essential. To investigate and control those phenomena, mechanism studies using a quartz crystal microbalance (QCM) system was performed to monitor thickness and mass variations. First, pure films of In_2_S_3_ and Cu_x_S were grown on Al_2_O_3_. While In_2_S_3_ had already been investigated with by in-situ QCM [26,27,28], the growth of ALD-Cu_x_S was investigated, to the best of our knowledge, for the first time with this set of precursors. Then, ion exchanges in the Cu-In-S ternary system were investigated by monitoring the growth of In_2_S_3_ on Cu_x_S and of Cu_x_S on In_2_S_3_ but also the behavior of the Cu precursor on In_2_S_3_. These have evidenced additional reaction paths that need to be controlled to pursue large scale CIS synthesis.

## 2. Materials and Methods

### 2.1. Thin Film Synthesis and In-Situ Growth Characterization

#### 2.1.1. Atomic Layer Deposition

In_2_S_3_ and Cu_x_S thin films were grown in a Beneq TFS-200 ALD reactor (15 × 15 cm^2^; Beneq Oy, Espoo, Finland), at T_dep_ = 180 °C for In_2_S_3_ and T_dep_ = 160 °C for Cu_x_S, respectively. High purity nitrogen 99.9999% (N_2_, Air Liquide, Paris, France) was used as carrier and purge gas. The carrier gas flow rate was set at 600 sccm. In the reaction chamber, the pressure was kept around 1 mbar. Indium(III) acetylacetonate (In(acac)_3_, 98%, Strem Chemicals, Bischheim, France), copper(II) acetylacetonate (Cu(acac)_2_, 98 + %, Strem Chemicals, Bischheim, France), and H_2_S (99.5%, Air Liquide, Paris, France) were used as In, Cu, and S sources, respectively. All chemicals were used as received, without further purification. A mass of 2 g of In(acac)_3_ and Cu(acac)_2_ was heated in two different hot solid sources (system Beneq HS300, Beneq Oy, Espoo, Finland) at T_In(acac)3_ = 150 °C and T_Cu(acac)2_ = 140 °C. To ensure proper mass transport of the metal precursors, “booster” and “combination” modes were chosen. In “booster” mode, N_2_ carrier gas is injected in the precursor source before its pulse. “Combined” mode is composed of five steps ([t_1_/t_2_/t_3_/t_4_/t_5_]). N_2_ is first injected in the source (t_1_, as in “booster” mode), all valves are kept closed (t_2_), precursor is pulsed (t_3_), and N_2_ is injected during the last part (t_4_) and after (t_5_) the precursor pulse. Typical growth cycles are {In_2_S_3_} = {[In(acac)_3_]/N_2_/H_2_S/N_2_ = [0.5/0.5]/5/0.5/5 s}, and {Cu_x_S} = {[Cu(acac)_2_]/N_2_/H_2_S/N_2_ = [0.5/0.2/0.5/10/0.5]/5/0.5/5 s}. A 10–50 nm thick Al_2_O_3_ passivation film was deposited on the reaction walls, prior to each experiment, and on the quartz crystal. It mitigates the cation exchange mechanisms [24,29], so that only the reactions between the quartz surface and the precursor(s) pulsed are revealed. Each experiment is referred in the text by the names given in the Table 1.

#### 2.1.2. In-Situ Quartz Crystal Microbalance (QCM) Measurements

In-situ QCM measurements were acquired with a Colnatec Eon-LT monitor system (Greenville, United States of America), using a HT quartz crystal covered by an alloy (6 MHz initial oscillation frequency, Neyco, Vanves, France) located downstream on the cover lid of the reactor. Signal was recorded every 0.2 s and the mass resolution set in order to have the lowest thickness step precision of 0.04 Å. Measurements were done after a long enough stabilization time to reach a uniform and constant temperature in the whole reaction chamber (ΔT ± 1.5 °C). Mass variations were calculated from the thickness values by considering the average density of the films grown by ALD as determined by X-ray Reflectivity (XRR) (4.75 g/cm^3^ for ALD-In_2_S_3_ and 5.52 g/cm^3^ for ALD-Cu_x_S). Impedance acoustic values were set at 0.27 (calculated from the shear modulus for a spray pyrolysis-In_2_S_3_ film obtained in the literature [30]) and 0.82 (considering the covellite phase), respectively.

### 2.2. Thin Film Characterization

Film thicknesses and densities were determined from samples deposited on glass substrates by X-ray Reflectivity using a PANalytical Empyrean equipment (PANanalytical, Orsay, France), equipped with a PIXcel 3D detector and Cu anode providing Cu-Kα radiations. Fitting of the experimental curves were performed with X’Pert Reflectivity Software (PANanalytical, Orsay, France). The morphology of the In_2_S_3_ samples was investigated with a field-emission scanning electron microscope (FESEM, Merlin VP Compact, ZEISS, Marly-Le-Roi, France). The electron beam acceleration voltage was set at 15 kV.

## 3. Results and Discussion

### 3.1. In-Situ Microgravimetric Study of In_2_S_3_ Material (Experiment #1)

**Figure 1:**
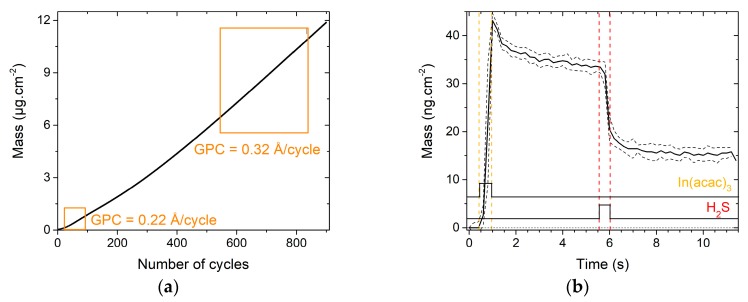
Mass variations during the deposition of In_2_S_3_ on a QCM covered by a 10 nm-thick Al_2_O_3_ layer; (**a**) mass variations all along the 900 cycles; (**b**) mass variations during one cycle averaged over ten cycles positioned in the linear portion of the curve (**a**). Vertical dash lines are a help for the eye to visualize the precursor pulse times.

Growth mechanism of In_2_S_3_ film was first studied and compared with the literature [26,27,28,31,32,33]. Figure 1a depicts the mass variation of In_2_S_3_ deposited on an Al_2_O_3_ substrate, monitored by the QCM during 900 cycles. The growth is non-linear as the estimated Growth Per Cycle (GPC) increases progressively until it reaches a constant value approximately equals to 0.32 ± 0.03 Å/cycle (determined by linear regression). This value is similar to what Genevée [31] (0.26 Å/cycle), Sterner [32] (0.30 Å/cycle), and Bugot [33] (0.24 Å/cycle) found using the same deposition temperature. The 400 first cycles, including the nucleation step, are characterized by a lower GPC, meaning that Al_2_O_3_ substrate partly inhibits the growth of In_2_S_3_, as observed by Sarkar et al. [27].

Figure 1b shows the mass evolution during a regular growth cycle of In_2_S_3_, i.e., of In_2_S_3_ on itself, averaged over 10 cycles. It is characterized by a mass gain during the In(acac)_3_ pulse (+(43 ± 2) ng.cm^−2^), a progressive mass loss during the purge (−(9 ± 1) ng.cm^−2^), and a mass loss during the H_2_S pulse (−(19 ± 1) ng.cm^−2^). These mechanisms correspond to the ligand exchange reactions, as explained elsewhere [27,28]. This description will serve as a reference for the study of ion exchanges with an ultrathin film of Cu_x_S.

### 3.2. In Situ Microgravimetric Study of Cu_x_S Material (Experiment #2)

**Figure 2:**
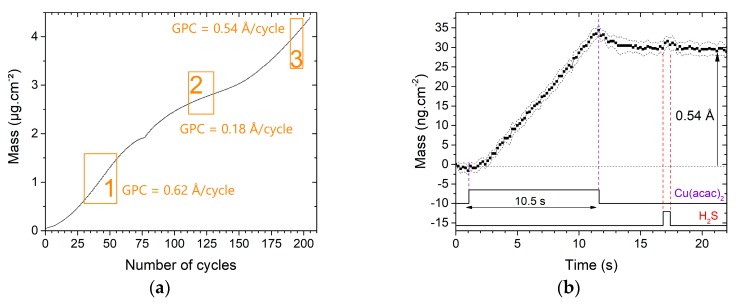
Growth mechanisms of Cu_x_S on a 50 nm-thick Al_2_O_3_ substrate; (**a**) mass variations all along the 200 cycles; (**b**) mass variations during one cycle averaged over ten cycles positioned in the last linear portion of the curve (rectangle number 3). Vertical dash lines are a help for the eye to visualize the precursor pulse times.

The growth mechanism of the second binary material in the Cu-In-S ternary system, i.e., Cu_x_S, was then investigated. The mass variation over 200 {Cu_x_S} deposition cycles is displayed in Figure 2a. The curve and thus the growth are non-linear, as the estimated GPC varies between low (0.1 Å/cycle) and relatively high values (0.60 Å/cycle). These variations all along the deposition may be partly explained by an instability of the flux, which has been observed during preliminary optimization efforts on Cu(acac)_2_ transport.

Figure 2b presents the mass evolution during one growth cycle of Cu_x_S on itself, averaged over 10 cycles (190th to 199th cycle). Estimated GPC equals 0.54 ± 0.03 Å/cycle. The mass slowly increases all along the pulse of Cu(acac)_2_ (+ 33 ng.cm^−2^ corresponding to + 0.6 Å for 10.5 s). The H_2_S pulse leads to a mass gain followed by a loss of similar value. Thus, this local variation has only a very small effect on the film mass gain.

Based on the theoretical surface chemical reactions that can be predicted and the mass (or thickness) variations obtained during the experimental reaction, it is possible to estimate which Cu_x_S phase has more chance to be deposited. According to Schneider et al. [34], the Cu_x_S synthesized by ALD with the precursors Cu(acac)_2_ and H_2_S may present a multiphasic state, that complicates the determination of the reactions enabling its synthesis.

Ideal reaction equations and mechanisms of the stoichiometric phases CuS (covellite) and Cu_2_S (chalcocite or digenite) can be hypothesized. For CuS, the overall reaction would be:

(1) $$\mathrm{Cu}{\left(\mathrm{acac}\right)}_{2}\;\left(g\right)+H_{2}S\left(g\right)\to \mathrm{CuS}\;\left(\mathrm{ads}\right)+2\;\mathrm{Hacac}\;\left(g\right),$$

which can be decomposed in two half-reactions during each Cu(acac)_2_ and H_2_S pulses (with “‖-“ depicting a surface group):

(2) $$\left||-\mathrm{SH}+\mathrm{Cu}{\left(\mathrm{acac}\right)}_{2}\;\left(g\right)\to |\right|-S-\mathrm{Cu}\left(\mathrm{acac}\right)+\mathrm{Hacac}\;\left(g\right),$$

(3) $$\left|\left|-S-\mathrm{Cu}\left(\mathrm{acac}\right)+{\;H}_{2}S\;\left(g\right)\to \right|\right|-S-\mathrm{Cu}-\mathrm{SH}+\mathrm{Hacac}\;\left(g\right).$$

The ratio of the reaction (R_s_) can then be calculated as follows:

(4) $$R_{s}=\frac{M\left(\mathrm{CuS}\right)}{M\left(\mathrm{Cu}{\left(\mathrm{acac}\right)}_{2}\right)-M\left(\mathrm{Hacac}\right)}=0.59.$$

For Cu_2_S, even though the ALD synthesis of this material has been reported by several groups [24,35,36,37], the detailed mechanisms is complex and, as far as we know, has never been investigated by QCM. The overall reaction may be:

(5) $$2\;\mathrm{Cu}{\left(\mathrm{acac}\right)}_{2}\;\left(g\right)+2{\;H}_{2}S\;\left(g\right)\to {\mathrm{Cu}}_{2}S\;\left(\mathrm{ads}\right)+4\;\mathrm{Hacac}\;\left(g\right)+S\;\left(g\right).$$

It is based on the assumption of the reduction of copper, from Cu^2+^ to Cu^+^, leading to a Cu-rich phase (chalcocite or digenite) from a Cu-poor phase (covellite) [34], accompanied with the oxidation and subsequent evaporation of sulfur:

(6) $$\left||-\left(2-x\right)\left[\mathrm{CuS}\right]\to |\right|-{\mathrm{Cu}}_{2-x}S+\left(1-x\right)S\;\left(s\right)\to ||-{\mathrm{Cu}}_{2-x}S+\left(1-x\right)S\;\left(g\right).$$

Though this equation is slightly endothermic (ΔG = 17 kJ/mol at 100 °C for x = 0 [38]), it is driven by the non-reversible evaporation of sulfur. The ratio of the reaction (R_s_) can be calculated as follows:

(7) $$R_{s}=\frac{M\left({\mathrm{Cu}}_{2}S\right)}{2M\left(\mathrm{Cu}{\left(\mathrm{acac}\right)}_{2}\right)-k\times M\left(\mathrm{Hacac}\right)-M\left(S\right)}=\frac{159.1}{523-k\times 100-32.1},$$

with k corresponding to the number of ligands desorbed during the Cu(acac)_2_ pulse, k = [0; 4]. As a function of k, R_s_ differs (see Table 2).

The reaction mechanisms and the phase formations can be discussed in light of the experimental results. According to the theoretical growth mechanisms estimated for the CuS formation, the desorption of ligands during the H_2_S pulse step should be characterized by a thickness drop (as M(acac) > M(SH)), which is not monitored by the QCM. The small variation during this H_2_S pulse may rather be characteristic of the formation of a Cu-rich phase. Indeed, a ratio of reaction of Cu_2_S close to 1, corresponding to an absence of thickness variation, can be determined for a certain value of k. We can estimate at 3.29 the number of ligands desorbed during the Cu(acac)_2_ pulse, while 0.71 ligands would be desorbed during the H_2_S pulse.

The growth mechanisms of Cu_x_S deposited on Al_2_O_3_ monitored by the QCM seem to be characteristic of a Cu-rich phase formation. They will serve as a reference for the microgravimetric study on the Cu-In-S ternary system.

### 3.3. Growth of CuInS_2_ Films (Experiments #3–5)

As described earlier, CIS thin film can be deposited by ALD by alternating cycles of In_2_S_3_ [26] and Cu_x_S [34] binary materials (supercycle strategy, [CIS] = n_1_ × {Cu_x_S} + n_2_ × {In_2_S_3_}), using β-diketonates precursors and H_2_S, at a temperature as low as 150 °C [16]. CIS stoichiometry was obtained only from some cycle ratios (n_2_:n_1_ > 5 at 150 °C), which demonstrated that there is a non-direct correlation between the cycle ratio and the film composition and evidenced side reactions that may compete with ALD traditional surface reactions. Indeed, as for other copper-containing sulfide materials [24,39,40], cation exchanges promoted by a rapid diffusion may occur during the CIS synthesis. This complexity has prevented a straightforward upscale of the CIS deposition in a large reaction chamber. To evidence cation exchanges in the Cu-In-S system and identify new paths to allow the deposition of large CIS films, growth of Cu_x_S and In_2_S_3_ on the other binary sulfide was investigated.

#### 3.3.1. Reaction Mechanisms of In_2_S_3_ on a Cu_x_S Substrate (Experiment #3)

**Figure 3:**
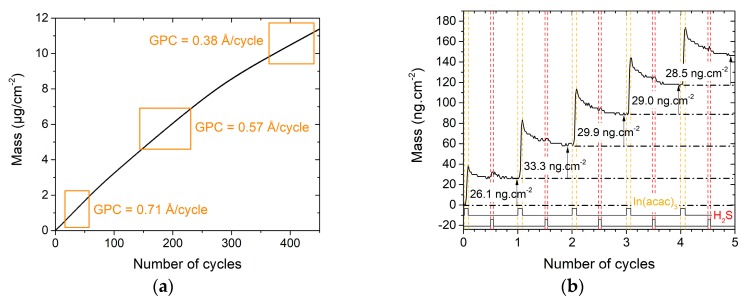
Mass variations during the deposition of In_2_S_3_ on a QCM covered by Cu_x_S (**a**) all along the 450 cycles; (**b**) zoom on the five first cycles.

Figure 3a shows the mass variation of an In_2_S_3_ film grown on a Cu_x_S substrate. It increases all along the 450 cycles, but the gain progressively slows down, with estimated GPC values from 0.71 Å/cycle for the first cycles to 0.38 Å/cycle for the last cycles (averaged values). It is characteristic of a substrate-enhanced growth that occurs over a large thickness. During the last cycles, after 20 nm is deposited, the GPC (0.38 Å/cycle) gets closer to the regular GPC of In_2_S_3_ (0.32 Å/cycle).

The first cycles were studied, in particular, as ion exchanges are more likely to occur during them. Thus, Figure 3b depicts the five first cycles of the growth and evidences the mass variation during each pulse and purge. The first In(acac)_3_ pulse leads to a lower mass gain than the following pulses, meaning that the surface of the substrate is less reactive or has a poorer density of absorption sites than the surface of the growing film. The H_2_S pulse is notably characterized by the absence or a weak mass variation during these first cycles, while a clear drop can be seen during the H_2_S pulse when In_2_S_3_ grows on Al_2_O_3_ (see Figure 1b). Two reasons might explain this behavior. First, the surface reaction of H_2_S gas may be inhibited by the substrate or the In(acac)_x_-terminated surface fragments. Second, the conversion from a Cu-rich phase to Cu-poor phase or covellite phase by copper dismutation may occur and some of the released sulfur remain at the surface to react with In(acac)_3_ (hence reducing the following mass gain during H_2_S pulse). No significant mass (or thickness) change has been observed during one of the precursor pulse, such as when diethyl zinc reacts on Cu_2_S [24], or when bis(N,N-disec-butylacetamidinato)dicopper(I) (CuAMD) reacts on Sb_2_S_3_ [40]. Therefore, there is no evidence that indium does readily ion exchange with copper in a Cu_x_S film at a deposition temperature of 180 °C.

**Figure 4:**
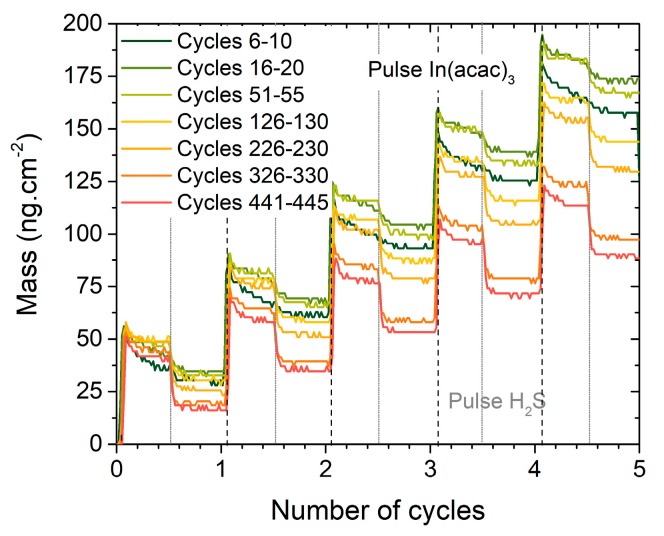
Aspect evolution of five successive cycles In(acac)_3_/N_2_/H_2_S/N_2_ distributed over the entire duration of the deposition, when the film grows on a Cu_x_S substrate.

To better understand the growth mechanism changes all along the deposition, first and last cycles are compared (Figure 4). Mass Gain Per Cycle (MGPC) is evidenced to vary and depends especially on the mass variation during the pulse and purge of H_2_S. Indeed, desorption during the H_2_S pulse steadily increases when the film grows, so that the MGPC decreases. As a consequence, the shape of the cycle is modified and finally appears to be similar to the cycle of growth of In_2_S_3_ on itself (see Figure 1b), which confirms that approximately 20 nm are necessary for the film not to be influenced by the substrate.

#### 3.3.2. Reaction Mechanisms of Cu_x_S on a In_2_S_3_ Substrate (Experiments #4–#6)

**Figure 5:**
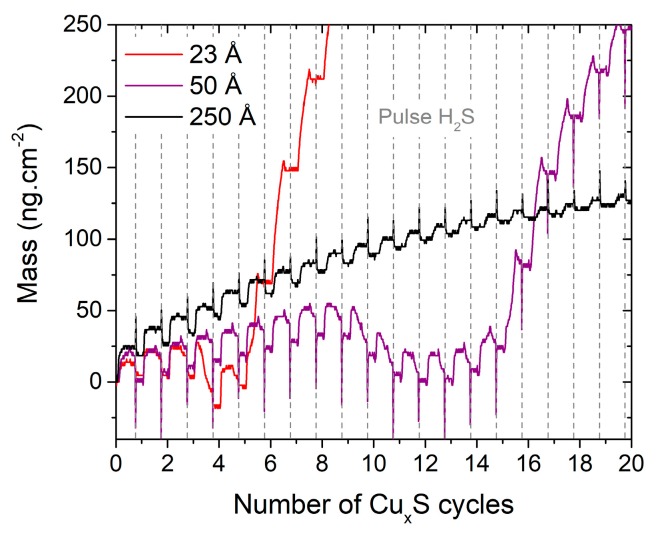
Mass variations measured for three films of In_2_S_3_ exposed to successive pulses of Cu(acac)_2_ and H_2_S. The apparent short-time mass changes observed during the H_2_S pulses (estimated thickness of In_2_S_3_ = 50 and 250 Å) are probably induced by temperature variation [41] and are considered as artefacts.

The reaction mechanisms occurring at the surface of a In_2_S_3_ substrate exposed to Cu(acac)_2_ and H_2_S pulses have been studied. Figure 5 shows the mass variations observed on In_2_S_3_ substrates of different thicknesses during the first cycles of Cu_x_S deposition. The curves are not superposed, which indicates that the exchange mechanisms differ as a function of the In_2_S_3_ thickness. For the thinner In_2_S_3_ substrates, the curves have non uniform variations and the mass decreases to a value lower than the initial one after several Cu_x_S cycles, then the film grows faster. Indeed, on 23 and 50 Å-thick In_2_S_3_ substrates, while most of Cu(acac)_2_ pulses show a mass gain, some depict large mass losses, respectively, the 4th and from the 10th to the 12th pulses. When the In_2_S_3_ substrate is thicker (250 Å), the curves have uniform variations.

**Figure 6:**
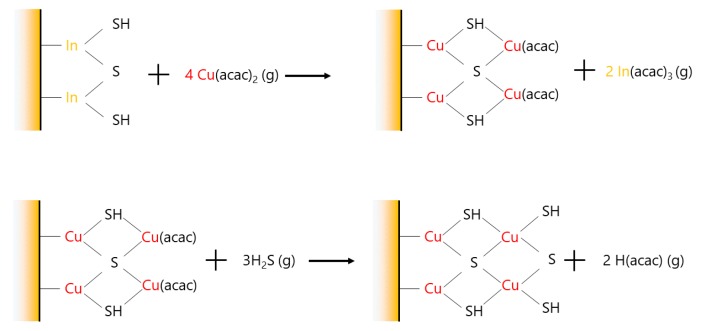
Exchange mechanism that would explain the mass loss during a H_2_S pulse.

Desorption phenomena have been described for multinary sulfide materials and are often linked to high cation mobility [8,42], coupled with fast diffusion of the cations as in ZnIn_x_S_y_ [28] or Cu(Zn,Sn)S_2_ (CZTS) [39]. Therefore, the large mass losses may be due to a gas-phase ion exchange between the Cu and In cations, from the precursor and the film, respectively. Heavy desorbed molecules (In(acac)_3_) would be replaced by lighter adsorbed molecules (Cu(acac)_2_), through an exchange mechanism that has to be determined. According to the shape of the curves in Figure 5, this process seems to slow down when the In_2_S_3_ film becomes thicker. As the mass loss does not occur during the first pulse, the reaction is not instantaneous but requires several cycles to be activated. In addition, unlike when Cu_x_S is grown on Al_2_O_3_ (Figure 2b), a mass drop occurs during most of the H_2_S pulses when Cu_x_S is grown on In_2_S_3_: first cycles for 23 or 50 Å-thick In_2_S_3_ and all along the cycles shown in Figure 5. After the mass loss, the shape of the cycle is modified because the mass variation during the H_2_S pulse vanishes. This mass drop occurring during the H_2_S pulse may be a consequence of the exchange mechanism as In(acac)_3_ molecules might be removed from the substrate as described by the reaction drawn in Figure 6. This hypothesis can be verified by considering the sign of the mass variations with the theoretical ones of Figure 6, i.e., $4\times M\left(Cu{\left(acac\right)}_{2}\right)-2\times M\left(In{\left(acac\right)}_{3}\right)>0$ during Cu(acac)_2_ pulse, $3\times M\left(H_{2}S\right)-2\times M\left(\mathrm{Hacac}\right)<0$ during H_2_S pulse, and $4\times M\left(\mathrm{Cu}{\left(\mathrm{acac}\right)}_{2}\right)-3\times M\left(\mathrm{Hacac}\right)-2\times M\left(\mathrm{In}{\left(\mathrm{acac}\right)}_{3}\right)>0$ for the global reaction. The recorded values: positive during the Cu(acac)_2_ pulse, negative during the H_2_S pulse and positive for the global reaction, verify the likeliness of the hypothesis. Further evidence would require more sophisticated experiment setup, possibly with gas phase species or in-situ surface composition characterization capabilities.

#### 3.3.3. Reaction Mechanisms of Cu(acac)_2_ on a In_2_S_3_ Substrate (Experiments #7 and #8)

**Figure 7:**
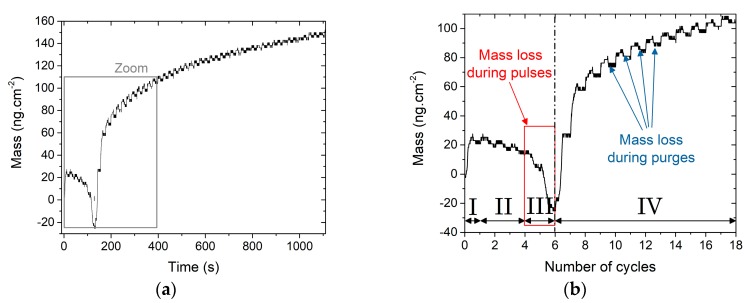
Growth mechanisms when Cu(acac)_2_ is successively pulsed on a 22 Å-thick In_2_S_3_ film, (**a**) mass variations during fifty successive pulses; (**b**) zoom on the first 18 pulses. The roman numbers correspond to the various growth phases.

**Figure 8:**
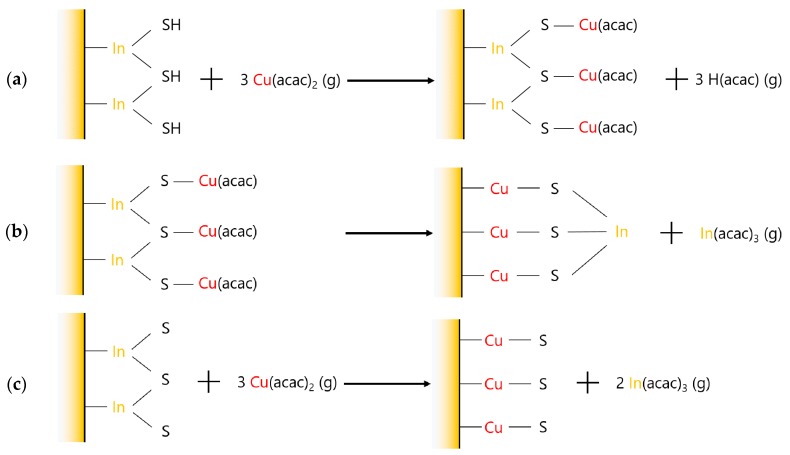
(**a**) Surface adsorption of Cu(acac)_x_ groups; (**b**) interdiffusion; (**c**) gas phase cation exchange mechanism.

The aim of these experiments was to determine if the Cu(acac)_2_ alone can activate the cationic exchange mechanism with the In atoms in the substrate. Compared to the {Cu_x_S} program, the H_2_S pulse is replaced by a purge, leading to the program [Cu(acac)_2_]/N_2_ = [0.5/0.2/0.5/10/0.5]/10.5 s. Figure 7a presents the complex variations of the mass during fifty successive pulses of Cu(acac)_2_ on a 22 Å–thick In_2_S_3_ film, with a zoom on the first 18 cycles in Figure 7b. A mass increase occurs during the first Cu(acac)_2_ pulse (+21.2 ng.cm^−2^), that may be ascribed to a surface adsorption of Cu(acac)_x_ groups on In_2_S_3_ substrate (phase I, described by the reaction in Figure 8a). During the three following cycles (phase II), the mass progressively decreases, especially during the purge time. Then (phase III, cycle 4 and 5), a mass loss is clearly visible during the pulse of Cu(acac)_2_ as it suddenly drops (down to −24 ng.cm^−2^). Finally, (phase IV), a mass gain is observed while Cu(acac)_2_ is pulsed, and this gain is progressively reduced during the following pulses, whereas a mass loss during the purges appears.

As described earlier, a desorption of In(acac)_3_ via an exchange mechanism is highly probable during phase III, as presented in Figure 8c, as it leads to a mass loss (3 × M(Cu(acac)_2_) − 2 × M(In(acac)_3_) < 0). Diffusion of Cu atoms along with a desorption of a small amount of In(acac)_3_ may explain the behavior during phase II (Figure 8b).

It is possible to determine if the In_2_S_3_ substrate is totally etched. The initial mass of the In_2_S_3_ substrate being 1040 ng.cm^−2^ for 22 Å corresponds to a mass of 733 ng.cm^−2^ of In (m(In) = m(In_2_S_3_) × 2M(In)/M(In_2_S_3_)), assuming no contaminants in the structure lattice. According to the Figure 8c process, as three Cu atoms replace two In atoms, a Cu mass equals to 647 ng.cm^−2^ would be necessary to replace all the In in the 22 Å-thick substrate (m(Cu) = [1.5 × m(In) × M(Cu)]/M(In)). This would be characterized by a mass loss equals to 125 ng.cm^−2^ while the experimental mass loss is 24 ng.cm^−2^ (end of phase III). It corresponds to only 19% of the mass loss estimated if all the In from the substrate was etched. Thus, it can be estimated than only 4.2 Å over the 22 Å (19%) are impacted by the cation exchange mechanism during the six first cycles of the experiment. In general, the surface saturation is improved by successive pulses of a precursor. For instance, Muneshwar and Cadien [43] have showed that in the case of ZrN and HfO_2_ depositions, using precursors with a high steric hindrance (Zr(NMe_2_)_4_ and Hf(NMe_2_)_4_), GPC was increased, respectively, ~46% and ~49% by reproducing at least 3 and 6 successive pulses. However, even when considering a similar steric hindrance for Cu(acac)_2_, it is surprising in our case to observe such a large mass increase (≈243%) over as much as 43 successive pulses. Hence, this confirms that additional phenomena, such as Cu diffusion, In substitution, and In desorption, occur until the 50th Cu(acac)_2_ pulse. The desorption of In, in particular, can explain the mass loss during the purges and liberates S atoms, newly available to bond with the adsorbed copper molecules.

**Figure 9:**
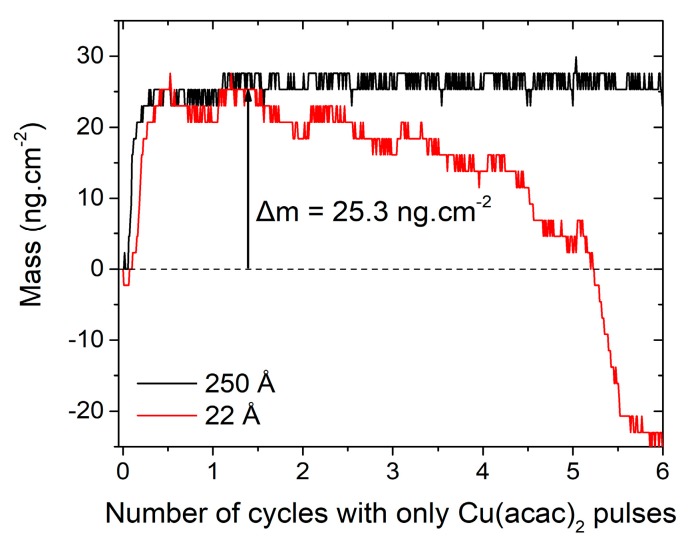
Mass variations recorded during six successive pulses of Cu(acac)_2_ on 22 Å- and 250 Å- thick In_2_S_3_.

**Figure 10:**
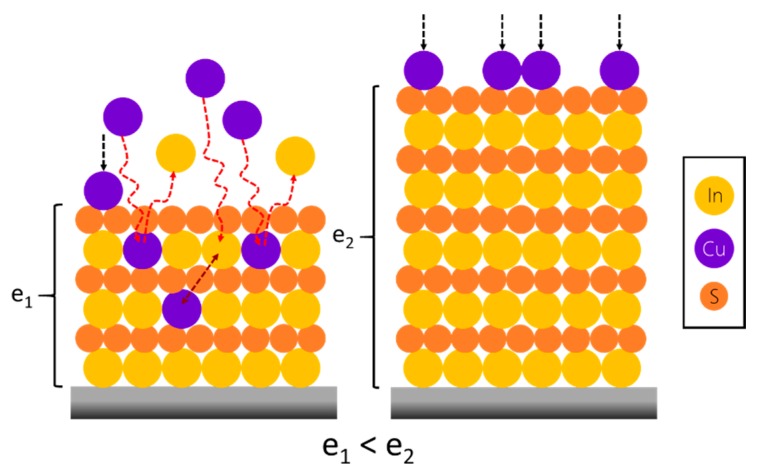
Possible reaction mechanisms between the Cu(acac)_2_ precursor in a gaseous state and a In_2_S_3_ film. When ultrathin (e_1_), In_2_S_3_ is more affected by ionic gas phase exchange and interdiffusion phenomena compared to a thicker film (e_2_).

**Figure 11:**
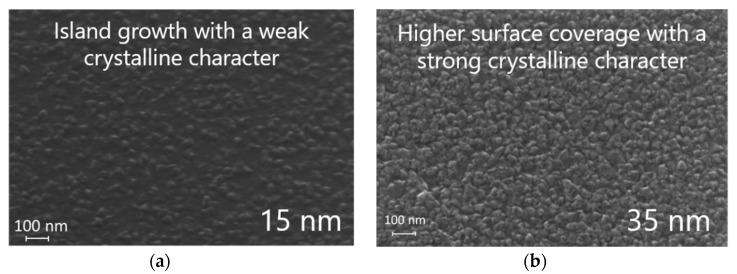
SEM images of In_2_S_3_ films tilted at 30°, (**a**) 15-nm thick; (**b**) 35-nm thick. Process program: In(acac)_3_/N_2_/H_2_S/N_2_: [0.5/0.5]/14/0.5/2.5 s; T_reactor_ = 180 °C.

To investigate the impact of the thickness on the growth, the same experiment has been conducted on a 250 Å-thick In_2_S_3_ substrate (experiment #8), and both are plotted in Figure 9. It shows that no mass loss is observed in the case of 250-Å thick substrate. This evidences that the growth and exchange mechanisms differ as a function of the In_2_S_3_ thickness, as represented Figure 10. This at first surprising observation can be explained by the impact of the In_2_S_3_ film morphology. Indeed, the morphology of the surface (Figure 11) and the crystalline character (as determined by XRD under grazing incidence, not shown here) of the film has been demonstrated to be dependent of the thickness. For the 15 nm-thick film, a Volmer-Weber growth mechanism can be considered to explain the island covered looking surface. For the thicker film, grains seem to cover the entire surface. Hence, it is possible that the cation exchange mechanism between Cu and In occurs preferentially on In_2_S_3_ islands that may have more reactive surface sites energetically favorable (Figure 5 and Figure 9).

**Figure 12:**
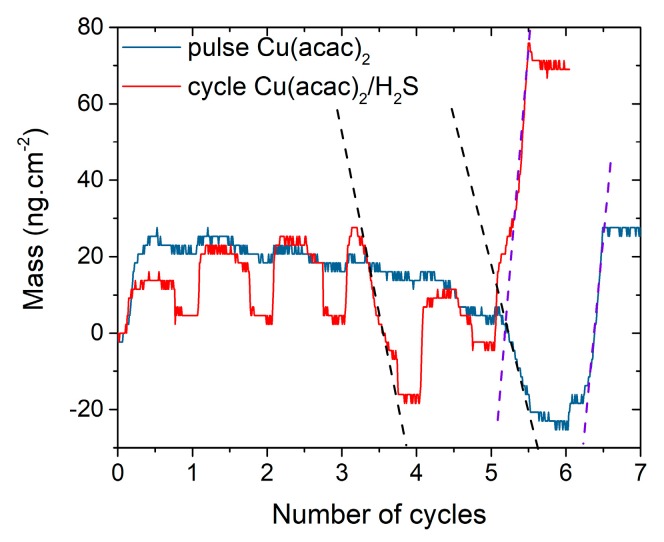
Mass variations observed during the first cycles of the reaction when a 22–23 Å- thick substrate is exposed to pulses of Cu(acac)_2_ alone or alternated pulses of Cu(acac)_2_ and H_2_S. The dashed lines represent the slopes of the curves.

Finally, the reaction of Cu(acac)_2_ alone or Cu(acac)_2_/H_2_S on a 23-Å thick In_2_S_3_ substrate are compared in Figure 12. The slopes of the mass loss during the Cu(acac)_2_ pulse and of the large mass gain that follows are similar whether H_2_S is used or not (see dashed lines on Figure 12). It suggests similar desorption and adsorption rates. The minimal mass values reached are also similar (−24 ng.cm^−2^ for Cu(acac)_2_, −18 ng.cm^−2^ for Cu(acac)_2_/H_2_S). In the latter case, about 14% of the In contained in the film is impacted by the exchange mechanism (3.2 Å over the 23 Å, for an initial mass = 1107 ng.cm^−2^) versus 19% for Cu(acac)_2_ alone. This difference can be explained by an enrichment of the film in sulfur, while H_2_S is pulsed, characterized by a mass loss and could be written as follows, with || representing a surface group:

(8) $$\left||-\mathrm{Cu}{\left(\mathrm{acac}\right)}_{x}+H_{2}S\;\left(g\right)\to |\right|-\mathrm{Cu}{\left(\mathrm{acac}\right)}_{x-1}\left(\mathrm{SH}\right)+\mathrm{Hacac}\;\left(g\right).$$

The desorption of acetylacetone during the first cycles would deprive the indium in the film of acac ligands and thus reduce the desorption of In(acac)_3_ during the fourth cycle. The H_2_S pulse seems to accelerate the cation exchange mechanism. Indeed, the mass loss phenomenon that occurs during a pulse of Cu(acac)_2_ happens two cycles earlier when H_2_S is used.

The nucleation behavior and especially the consequence of the first Cu(acac)_2_ exposure seems also to differ. To estimate the coverage of Cu(acac)_2_ species, the number of In_2_S_3_ surface sites can be approximated by using the In_2_S_3_ average density (4.75 g/cm^3^ as determined by XRR, equivalent to ρ = 8.8 × 10^21^ “In_2_S_3_ units”/cm^3^), and assuming a square lattice, leading to ρ^2/3^ = 4.3 × 10^14^ “In_2_S_3_ units”/cm^2^.

The coverage of Cu(acac)_2_ can then be approximated based on the mass gain during the first exposure. In the case of the experiment using only Cu(acac)_2_ precursor, 21.2 ng/cm^2^ is deposited that corresponds to 4.88 × 10^13^ “Cu(acac)_2_ molecules”/cm^2^. The normalized coverage of Cu(acac)_2_ species relative to “In_2_S_3_ units” on the surface is [4.88 × 10^13^ “Cu(acac)_2_ molecules”/cm^2^]/[4.3 × 10^14^ “In_2_S_3_ units”/cm^2^] = 0.11 “Cu(acac)_2_ molecules”/“In_2_S_3_ units”, i.e., a coverage of 11%. In the case of alternated Cu(acac)_2_ and H_2_S precursor, a mass gain of 12.9 g/cm^2^ is obtained, that yields a coverage of 7%, based on similar assumptions. These coverage values are reasonable, as they are significantly smaller than the maximum coverage calculated to be 180% ([7.76 × 10^14^ “Cu(acac)_2_ molecules”/cm^2^]/[4.3 × 10^14^ “In_2_S_3_ units”/cm^2^]) according to the steric hindrance of the Cu(acac)_2_ molecules (see Appendix A). As the same Cu(acac)_2_ pulse time is used, the coverage is indeed similar, and the observed small variations may be due to slightly different In_2_S_3_ initial surface state.

## 4. Conclusions

This study is a deep investigation of the reaction mechanism and ion exchanges occurring in the ternary Cu-In-S system prepared by ALD from metal acetylacetonate precursors and H_2_S. This was performed using an in-situ quartz crystal microbalance system. For this, growth of In_2_S_3_ and Cu_x_S binary compounds on a Al_2_O_3_ substrate were first studied. It strengthened the postulated mechanism for the ALD-Cu_x_S formation via reduction of Cu, oxidation and subsequent evaporation of elemental sulfur. Then, mass variations were monitored during the synthesis of In_2_S_3_ on a Cu_x_S substrate, of Cu_x_S on a In_2_S_3_ substrate, and pulsing Cu(acac)_2_ on a In_2_S_3_ substrate. Mass variations have evidenced gas-phase ion exchanges when Cu(acac)_2_ reacts on an In_2_S_3_ substrate while In_2_S_3_ growth is enhanced by a Cu_x_S substrate. It was particularly highlighted by a large mass loss that occurs during one or two of the first Cu(acac)_2_ pulses. It has also been noted that it may be possible to reduce the cation exchange by controlling precisely the In_2_S_3_ thickness. Indeed, from a certain thickness value (and morphology), the reaction of Cu(acac)_2_ on a In_2_S_3_ substrate is even hindered, as in our case from 250 Å. Indeed, the crystalline character of In_2_S_3_ is enhanced by the thickness, and it was shown to be more stable when Cu(acac)_2_ reacts on its fully covered surface rather on growth islands. To go further, analytical tools, such as XPS (ex-situ or in-depth) or in-situ XRF, may be employed in order to determine precisely how deep the In_2_S_3_ film is impacted by the exchange mechanism (in-depth In and Cu concentration) as a function of the number of cycles of Cu_x_S. In addition, Quadrupole Mass Spectrometry measurements could help to identify the reaction by–products.

Finally, beyond the fundamental interest in understanding the ever-challenging growth of ALD multinary compounds, it also aims at allowing the upscale of an ALD recipe. Indeed, it explains why the film synthesized during the CIS deposition in the Beneq TFS-200 is not homogeneous and does not contain any indium upstream, next to the gas inlet.

Based on these observations, a nanolaminate strategy that controls finely the In_2_S_3_ thickness deposited between each Cu_x_S cycle, should rather be chosen to deposit homogeneous CIS film on large area. Another solution would be to substitute one of the metal precursor by a precursor without acac ligands as this may lead to different dynamics.

## Figures and Tables

**Table 1:** In situ microgravimetric experiments between precursors Cu(acac)_2_-In(acac)_3_-H_2_S and the sulfide materials In_2_S_3_ and Cu_x_S initially deposited on the quartz crystal microbalance (QCM).

Experiment	Substrate (Estimated Thickness)	PRECURSOR(s) Pulsed Sequentially	T_REACTOR_
#1	Al_2_O_3_ (100 Å)	In(acac)_3_/H_2_S	180 °C
#2	Al_2_O_3_ (500 Å)	Cu(acac)_2_/H_2_S	160 °C
#3	Cu_x_S (95 Å)	In(acac)_3_/H_2_S	180 °C
#4	In_2_S_3_ (23 Å)	Cu(acac)_2_/H_2_S	160 °C
#5	In_2_S_3_ (50 Å)	Cu(acac)_2_/H_2_S	160 °C
#6	In_2_S_3_ (250 Å)	Cu(acac)_2_/H_2_S	160 °C
#7	In_2_S_3_ (22 Å)	Cu(acac)_2_	160 °C
#8	In_2_S_3_ (250 Å)	Cu(acac)_2_	160 °C

**Table 2:** Theoretical ratios (R_s_) of the overall reaction for the formation of Cu_2_S, as a function of k, the number of ligand desorbed during each Cu(acac)_2_ pulse.

k	0	1	2	3	4
**R_s_**	0.32	0.41	0.55	0.83	1.75

## Appendix A

According to its crystal structure, the area of a Cu(acac)_2_ molecule can be estimated to be similar to a rectangle, with a length of 11.1 Å (distance between C(4A) and C(5)) and a width of 4.05 Å (distance between C(4) and C(5)) [44]. Thus, the calculated area would be 44.7 Å^2^. When adsorbed, the complex loses a ligand and it binds through the Cu atom with a molecular axis being perpendicular to the surface as reported by Hu et al. for an adsorption on Ta(110) surface [45]. Its projection on the In_2_S_3_ surface can be modelled as a circle with a diameter of 4.05 Å, representing a surface area of 12.9 Å^2^. The maximum areal density of the adsorbed Cu(acac)_2_ molecules would then be equal to 7.76.10^14^ Cu(acac)_2_/cm^2^.
